# A resampling-based approach to share reference panels

**DOI:** 10.1038/s43588-024-00630-7

**Published:** 2024-05-14

**Authors:** Théo Cavinato, Simone Rubinacci, Anna-Sapfo Malaspinas, Olivier Delaneau

**Affiliations:** 1 Department of Computational Biology, University of Lausanne, Lausanne, Switzerland; 2 Swiss Institute of Bioinformatics, University of Lausanne, Lausanne, Switzerland; 3 Division of Genetics, Department of Medicine, Brigham and Women’s Hospital and Harvard Medical School, Boston, MA USA; 4 Program in Medical and Population Genetics, Broad Institute of MIT and Harvard, Cambridge, MA USA; 5 Regeneron Genetics Center, Tarrytown, NY USA

**Keywords:** Haplotypes, DNA sequencing, Genome-wide association studies

## Abstract

For many genome-wide association studies, imputing genotypes from a haplotype reference panel is a necessary step. Over the past 15 years, reference panels have become larger and more diverse, leading to improvements in imputation accuracy. However, the latest generation of reference panels is subject to restrictions on data sharing due to concerns about privacy, limiting their usefulness for genotype imputation. In this context, here we propose RESHAPE, a method that employs a recombination Poisson process on a reference panel to simulate the genomes of hypothetical descendants after multiple generations. This data transformation helps to protect against re-identification threats and preserves data attributes, such as linkage disequilibrium patterns and, to some degree, identity-by-descent sharing, allowing for genotype imputation. Our experiments on gold-standard datasets show that simulated descendants up to eight generations can serve as reference panels without substantially reducing genotype imputation accuracy.

## Abstract (web summary)

The authors develop the tool RESHAPE to share reference panels in a safer way. The genome–phenome links in reference panels can generate re-identification threats and RESHAPE breaks these links by shuffling haplotypes while preserving imputation accuracy.

## Main

Genotype imputation is the statistical estimation of missing genotypes in an SNP (single nucleotide polymorphism) array data, using a reference panel of sequenced individuals. Genotype imputation is ubiquitous in the field of statistical genetics and genome-wide association studies (GWAS), as it drastically increases the number of genetic variants available, which helps boost association signals, identify causal variants and meta-analyze multiple cohorts^1^. Genotype imputation predicts missing data in a target sample by considering target haplotypes as mosaics of reference haplotypes^2–4^. The most commonly used imputation model is based on the Li and Stephens hidden Markov model^5^ that probabilistically builds haplotype mosaics in agreement with the variable recombination rate in hotspots and coldspots observed in the human genome^6^. In the past 10 years, the size of the reference panels used for genotype imputation has increased considerably, improving the accuracy of genotype imputation, in particular at rare variations. This has been possible thanks to the establishment of large-scale projects such as the 1000 Genomes Project (1000GP)^7^, the Haplotype Reference Consortium (HRC)^8^, the TOPMed program^9^ and, more recently, the UK Biobank resource^10–12^. However, reference panels part of nationwide biobanks, with sample sizes on the order of hundreds of thousands of genomes, comprise sample-level genetic and phenotypic data linked together, which puts strict restrictions on accessibility and data sharing and therefore prevents their wide usage for genotype imputation in other studies. In this work, we present RESHAPE (Recombine and Share Haplotypes), a method that enables the generation of a synthetic haplotype reference panel by simulating hypothetical descendants of reference panel samples after a user-defined number of meiosis. This method is conceptually similar to the algorithms implemented in HAPGEN^13,14^ and HAPNEST^15^, tools that generate synthetic reference panels to assess the effect of different genotyping chips on GWAS power or to compare polygenic risk scoring (PRS) methods. The reference panels generated by RESHAPE allow for high-quality genotype imputation while (1) mixing the genetic data across samples, (2) disrupting genome–phenome links, and (3) preventing the usual re-identification threats faced by anonymized reference panels^16^. We assessed the impact of our approach on imputation accuracy using multiple gold-standard reference panels, varying the number of generations used to simulate descendants. Our approach aims to facilitate reference panel sharing by proposing an alternative dataset that is still useful for imputation and can be made available under less restrictive data-sharing policies.

## Results

### Model overview

**Fig. 1:**
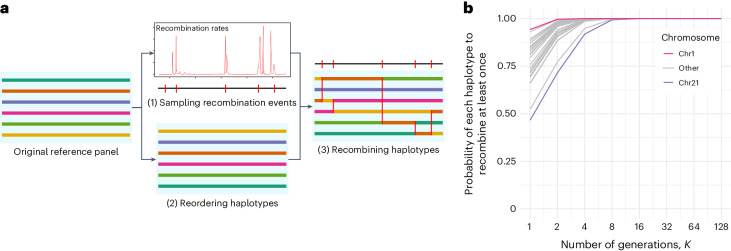
Description of the method. **a**, The method takes a reference panel as input and (1) samples recombination sites using a Poisson process and a genetic map, (2) randomly reorders the haplotypes and (3) recombines the haplotypes based on the drawn recombination sites, resulting in a synthetic reference panel. **b**, Probability of each haplotype of the reference panel to recombine at least once using our approach for different chromosome size in centimorgans. Each of the 22 lines correspond to the result for a given chromosome size in centimorgans. The blue line corresponds to the largest chromosome (chromosome 1, 286.28 cM) and the red line corresponds to the smallest chromosome (chromosome 21, 62.79 cM).

Through meiosis, the chromosomes from a parent recombine, thereby creating new haplotypes that will be transmitted to the offspring. This process is repeated at each generation, involving that an individual’s haplotypes can be represented as mosaics of haplotypes of its ancestors. The method we propose in this work builds on this idea and consists of two separate steps. First, it samples recombination points in the genome based on a genetic map and a user-defined number **K** of simulated meiosis. Second, it recombines reference haplotypes at these positions to generate a given number of offspring haplotypes in which the linkage disequilibrium (LD) structure and, to some extent, identity-by-descent (IBD) sharing are preserved (Fig. 1a). The resulting synthetic reference panel is of the same size as the original, maintains the necessary information for the Li and Stephens^5^ imputation model, and disrupts the association of genotypes with individuals and therefore with any individual phenotypic data. Sharing a synthetic reference panel can be seen as distributing the haplotypes of relatively distant relatives of the original samples included in the reference panel.

### Impact of the simulated recombination process on LD

**Fig. 2:**
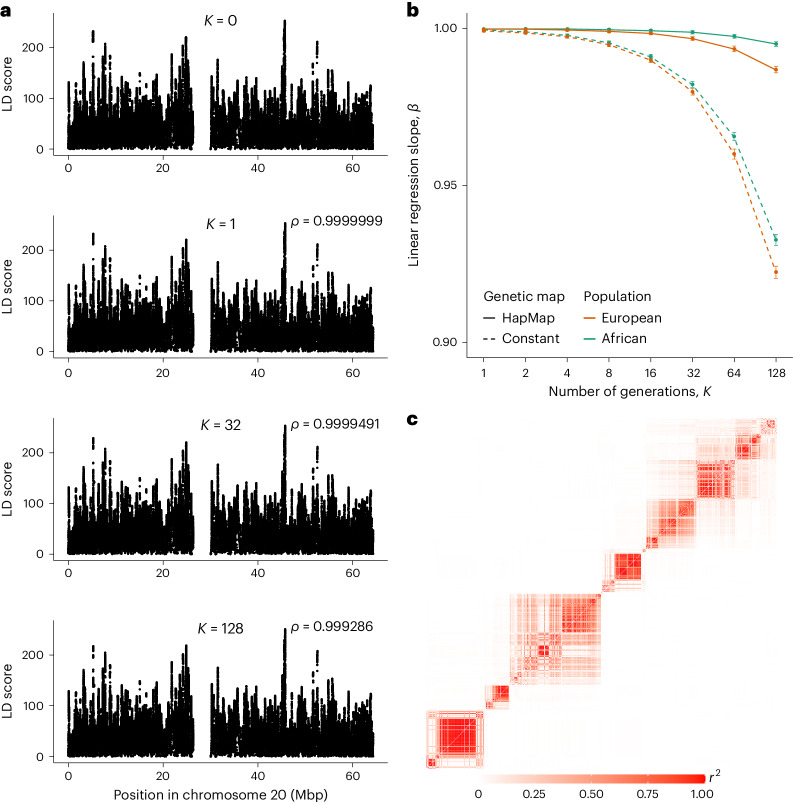
LD score of synthetic haplotypes. **a**, Average LD score through 50 replicates of the European reference panel generated using *K* = 0, 1, 32 and 128. The value *ρ* is the Pearson correlation coefficient between LD scores obtained for *K* = 0 and LD scores obtained for *K* = 1, *K* = 32 and *K* = 128. **b**, Average linear-regression slope (*β*) between the original reference panel and synthetic reference panels through 50 replicates (*y* axis) for different values of *K* (*x* axis). Colors correspond to the population analyzed (European, orange; African, green). The error bars correspond to the mean of the 50 replicates ± s.d. **c**, Correlation (*r*^2^) between SNPs in a 300-kbp-long region of chromosome 20. Values above the diagonal were obtained from the original European reference panel and values under the diagonal were obtained from a synthetic European reference panel generated using *K* = 128. Source data

We investigated the effect of the number of generations (*K*) on LD scores in two populations: the European and African samples of the 1000GP. Overall, we found that our recombination approach has a low impact on LD scores, even with a high number of generations (that is *K* = 128), and maintains high correlation levels with the original LD scores (Fig. 2a). We, however, observed a general decline in LD scores, as expected by the introduction of new recombination events in the data (Fig. 2b). This decline is far more pronounced when using a constant-rate genetic map than with a HapMap genetic map, consistent with the more frequent occurrence of highly LD disruptive recombination events in recombination coldspots when assuming a constant recombination rate. For both HapMap and constant recombination maps, LD scores for the African population are slightly less affected than for the European population, probably due to the overall lower LD in this population. Finally, closer inspection of pairwise LD values revealed that the fine LD structure is remarkably well preserved even when using a high number of generations (Fig. 2c).

### Impact on imputation accuracy

**Fig. 3:**
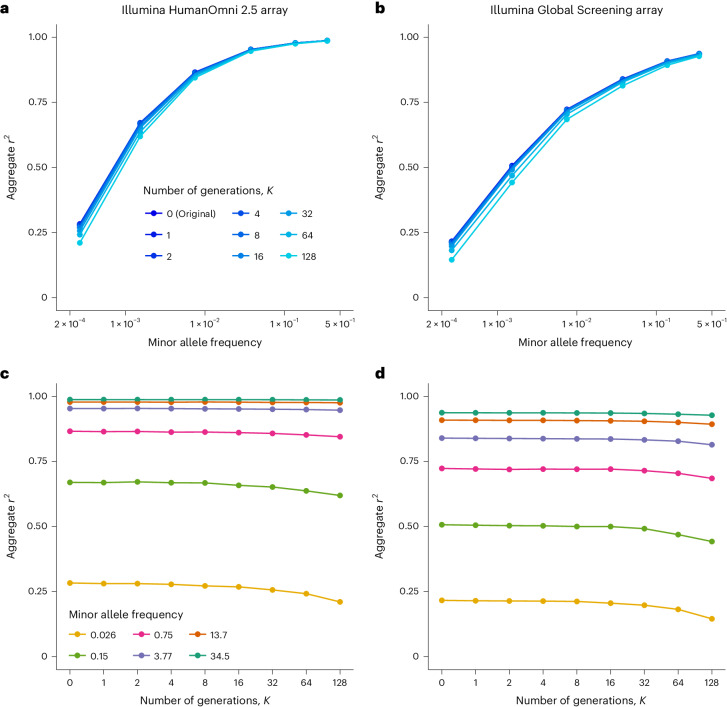
Imputation accuracy on synthetic haplotypes from the 1000GP. Aggregate *r*^2^ depending on the number of generations used to recombine the haplotypes. **a**,**b**, Shades of blue corresponds to the values of *K* used to recombine the haplotypes. **c**,**d**, Colors correspond to minor allele frequency bins. Results with the Illumina HumanOmni 2.5 array (**a**,**c**) and the Illumina Global Screening array (**b**,**d**) are shown. Source data

**Fig. 4:**
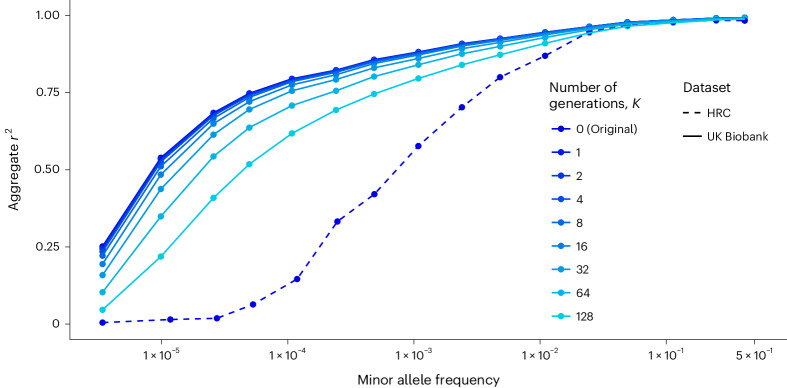
Imputation accuracy on synthetic haplotypes from the UK Biobank. Aggregate *r*^2^ depending on the number of generations used to recombine the haplotypes (shades of blue). Dashed and solid lines distinguish results obtained with the HRC from the results obtained with the UK Biobank. Source data

Having established that LD is well preserved, we then assessed the extent to which recombining haplotypes in a reference panel affects genotype imputation accuracy. To do so, we imputed genotypes in 52 individuals from the 1000GP (2 individuals from each population) using the remaining 2,452 samples as reference panels, either recombined at different levels (*K* = 1, 2, 4, 8, 16, 32, 64 or 128) or not (*K* = 0). Overall, we find that our recombination approach with up to *K* = 8 has a low impact on imputation and maintains a decrease in imputation accuracy below 0.01, regardless of the SNP array used (Fig. 3a,b). Moreover, the difference in imputation accuracy between *K* = 0 and *K* = 8 remains small regardless of the mixed/non-mixed ancestry of the target individual (Supplementary Fig. 5). Further recombining the data up to *K* = 128 leads to a moderate decrease of 0.07 in imputation accuracy. Importantly, we find that rare variants seem to be more affected by the recombination procedure than common variants, for which almost no loss of accuracy is observed (Fig. 3c,d). To further investigate this effect on rare variants, we used the sequencing data for 147,754 UK Biobank samples in our imputation experiments. We split the dataset in 1,000 target and 146,754 reference samples, generated synthetic reference panels with various numbers of generations and used the resulting data to impute the target samples. We find similar patterns to the 1000GP data: common variants are imputed well regardless of the number of generations whereas rare variants are more affected (Fig. 4). The largest drops in imputation accuracy are reached for extremely rare variants with a minor allele frequency <0.005% (1 haplotype out of 20,000 carries a copy of the minor allele), with an *r*^2^ decreasing by 0.03, 0.10 and 0.32 for *K* = 8, *K* = 32 and *K* = 128, respectively. Two important observations can be made here. First, the drop in imputation accuracy remains below 0.05 for a number of generations up to *K* = 8, suggesting that *K* = 8 maintains high imputation accuracy even in very large reference panels. Second, the important loss of accuracy observed at *K* = 128 still provides better imputation accuracy than the not-recombined HRC panel^8^, which contains fewer haplotypes but is accessible under less restrictive data-sharing policies than the UK Biobank.

### Comparison with HAPGEN and HAPNEST

Similar approaches consisting of sampling real genetic data to generate in silico datasets have been implemented in HAPGEN and HAPNEST: tools that simulate individual-level genotypic and phenotypic data to assess the effect of different genotyping chips on GWAS power or to compare PRS methods. However, these are not specifically intended for the simulation of synthetic reference panels suitable for imputation purposes. One of the main differences between RESHAPE and these two approaches is the fact that when producing synthetic haplotypes, HAPGEN and HAPNEST do not necessarily sample from all of the reference haplotypes and from each of their positions, which might result in a loss of genetic information. This has two important consequences: (1) their approach cannot be reversed as some of the information present in the original reference panel are lost and (2) some IBD segments that could have been shared with a sample to impute might be lost, which result in a decrease in imputation accuracy. Nonetheless, we were curious to evaluate their performance compared with our proposed approach. Thus, we conducted a comparative analysis of RESHAPE, HAPGEN and HAPNEST in terms of speed and memory consumption using datasets with varying numbers of samples and variants (Supplementary Methods).

Our findings demonstrate that RESHAPE substantially outperforms the other algorithms in terms of memory consumption across all scenarios, showing an improvement in the order of magnitude (Supplementary Fig. 1). In addition, while the speed of all algorithms decreases with an increase in the number of samples or variants, RESHAPE remains faster than the other algorithms. Moreover, HAPGEN and HAPNEST require a pre-processing of the original reference panel that we did not take in account in our comparisons, but which strongly adds to the time required to run the full process. The observed disparities in performance may be attributed to the varying design of the algorithms. Unlike HAPGEN and HAPNEST which, based on our results, seem to store the entire reference panel in memory before modifications and writing, RESHAPE adopts an efficient streaming process, modifying and writing one line of the reference panel at a time.

Furthermore, we investigated the impact of these approaches on imputation accuracy (Supplementary Fig. 2). Due to the absence of phased VCF (variant call format) output from HAPNEST, our comparison was limited to HAPGEN. It is worth mentioning that a future release of HAPNEST may include this feature, which prompted us to include it in the speed and memory comparisons. RESHAPE using *K* = 8 and *K* = 128 outperforms the output of HAPGEN. This may be due to the loss of important IBD segments as previously explained, or to a very high shuffling level of the HAPGEN algorithm which would have disrupted important IBD fragments.

## Discussion

We presented RESHAPE, an approach to remove genome–phenome links in a reference panel while maintaining key features necessary for genotype imputation. There is a trade-off between the level at which RESHAPE recombines the reference haplotypes and the conservation of LD structure and imputation accuracy: these two properties decay with *K*, but the higher the *K* and the higher the chance of all haplotypes to recombine at least once. We have shown that *K* = 8 is a reasonable solution as (1) the probability of all haplotypes to recombine at least once is above 0.99, (2) the LD structure is highly conserved and (3) imputation accuracy is comparable to imputation accuracy obtained with the original reference panel. If we assume that 8 generations equate to approximately 200 years, then the sharing of a synthetic reference panel generated using *K* = 8 could be compared as though people living under George IV’s reign had access to genomes of their present-day descendants (in the special scenario where non alleles of the founders are lost). While the use of a constant-rate genetic map only slightly affects imputation accuracy for *K* ≤ 8 (Supplementary Fig. 3), using a constant-rate genetic map could facilitate the identification of the sampled recombination sites and therefore may enable the reconstruction of the original haplotype. Moreover, genetic maps have a stronger effect for superior values of *K* and we thus encourage users to use a genetic map derived from a population close to the population present in their reference panel when possible.

With a sufficiently high number of generations provided as input, RESHAPE removes genome–phenome links, which goes one step further than anonymization against re-identification threats in reference panels. In a reference panel where no information is linked to each pair of haplotypes, one could still infer phenotypes from an haplotype pair to deduce the identity of its donor. This re-identification by phenotypic prediction relies on the fact that phenome inference narrows down the potential pool of genome owners^17^. For instance, in a biobank where genomes were gathered across the same country, each genome has an equal probability of belonging to any resident of that country. Using metadata of the cohort, for example, the fact that participants are in a certain age range, one can narrow down the pool of possible genome donors. Complete identification is achieved when this narrowed-down number equals one. Inferring the phenotypic traits of each member of a reference panel would add to the metadata linked to each genome, and thus help to re-identify its owner. Recombining haplotype fragments disrupt the phenome carried by the original haplotypes, thereby avoiding re-identification by phenotypic prediction. This approach could be particularly useful in the current context where the biobank-scale reference panels are widening every year but remain under strongly restrictive data-sharing policies.

In this paper, we focused on the effect of our method on imputation. While a synthetic reference panel conserves LD structure and allele frequencies, it might not be suitable as input for other approaches relying on these properties because of the assumptions we made in our model. First, we do not consider crossover interference, which results in simulated recombination events that do not fully reproduce the natural recombination process. Moreover, males and females show different recombination rates and we here use only one genetic map as input. Future users that would use synthetic reference panels for other purposes should first assess the effect of our assumptions on their results. Nevertheless, depending on the future requirements of our users, we could incorporate additional features that can accommodate these particular processes in a new version of RESHAPE. While some characteristics might not be conserved by our transformation, the original reference panel can be easily restored from the synthetic one when knowing the parameters that were used for the transformation. This characteristic has the potential to be useful in the sharing of data, but it falls outside of the scope of this paper’s intended focus.

We anticipate that it may be possible to use haplotype-sharing techniques to detect the presence of a specific individual or their relative in a synthetic reference panel. For instance, long IBD segments shared between a target individual and a synthetic reference panel can strongly suggest that the target individual or one of their close relatives is present in that panel. However, the presence of an individual in a reference panel would only raise privacy issues if the participation in the reference panels cohort results in sensible attribute disclosures, for example, if all the participants share a sensitive disease. Otherwise, the synthetic reference panel could be used only to retrieve a larger amount of genotypic data of the target individual, which could also be obtained by imputation of this sample with another reference panel. Finally, these approaches require access to the genotype data of an individual included in the reference panel, which is typically unlikely. The disclosure of extra information about the original reference panel may pose another potential threat, as it may facilitate the recovery of the original panel from a synthetic one without knowledge of the parameters used in its creation. One approach could rely on a leak of phenotypic data. In this case, recovering the original panel would entail finding the mosaics of synthetic haplotypes that best explain (based on PRS) the known phenotypes. However, we anticipate that this problem would be extremely difficult to solve in practice without any guarantee of restoring the original data. Another attack could rely on the availability of SNP array data for the original samples. In this scenario, imputing the SNP array data using the synthetic reference panel should produce genetic data close to the original panel. However, imputing the SNP array data with another large-scale reference panel would lead to a similar outcome.

With the availability of modern reference panels, several new challenges need to be addressed using different strategies and approaches. One such approach is meta-imputation^18^, which aims to leverage multiple imputation servers based on different reference panels to accurately impute target samples. Another approach is imputation using homomorphic encryption^19^, which aims to protect sensitive data on the user side by encrypting target genotypes before sending them to imputation servers. However, this approach is still restricted to linear models, which are known to perform poorly on rare variants; the main benefit of using large reference panels. To complement this toolbox, we suggest a technique for generating a synthetic reference panel that retains imputation information while removing the original genome–phenome association. This could be advantageous for both centralized and local imputation servers, as the synthetic panels would no longer contain the most sensitive information, potentially increasing the willingness of large consortia to share their reference data for imputation purposes.

## Methods

### Algorithm

Let N and L be the number of haplotypes and variant sites in the reference panel. Naive implementation of the scheme described above would involve multiple forward simulations in which a new synthetic reference panel is created at each generation. The computational cost in this case would be proportional to *O*(KNL). In our work, we propose an alternative two-step approach, which has the advantage of involving a computational cost proportional to *O*(NL), that is, essentially independent of K, the number of meiosis.

In the first step, we model the total number of recombination events in a specific genomic region as a Poisson distribution with **λ** equal to the average number of recombination sites expected in that region, that is, the size of this region in Morgan times (N/2)K, to account for the number of generations simulated and the number of haplotypes in the dataset. Thus, we can simulate genomic positions at which recombination events occur along a chromosome using a Poisson process, which consists of drawing genetic distances in centimorgans (cM) from the inverse cumulative distribution function (CDF^−1^) of a Poisson distribution. This results in an array **R** = {r_1_, …, r_M_} of genomic positions r with M being the total number of recombination events drawn. Finally, all these genetic positions in centimorgans are converted into base pairs (bp) using the genetic maps provided as input.

In the second step, we start by shuffling the indexes of the reference haplotypes to establish an initial random order. Then, we stream the haplotype data in an output file according to this new order for all haplotypes at every site until we reach the first recombination event in **R**. When this happens, we update the current ordering by permuting (recombining) two randomly sampled haplotypes (with replacement) and we carry on streaming the data until we reach the next recombination event. We repeat this procedure until the end of the chromosome, permuting two haplotypes each time we encounter another recombination event. These two steps are described in more detail in the form of a pseudocode in Supplementary Information (Supplementary Fig. 4).

The synthetic reference panel comprises the same number of haplotypes and variant sites as the original dataset. This approach has two important properties. First, the data transformation is straightforward to reverse. Indeed, we can easily regenerate the full sequence of events leading to the synthetic data knowing the genetic map, the value of *K* and the seed of the random number generator, and therefore restore the data to its original state. Second, some haplotypes may not recombine. The probability that a given haplotype recombines at least once can be derived from a Poisson distribution and increases with *K* and the length in centimorgans of the chromosome (Fig. 1b). Thus, with *K* ≥ 8, even the smallest chromosome (chromosome 21, 62.79 cM) has a probability of recombining at least once that exceeds 0.99.

We implemented our procedure and its reverse in a C++ software called RESHAPE. By providing it with a genetic map and the VCF/BCF (binary variant call format) of a reference panel, RESHAPE outputs a VCF/BCF of the same size containing descendant haplotypes.

### Benchmark

In our experiments, we used phased data from two well-known reference panels of haplotypes: (1) the 1000GP (ref. ^7^) comprising data for 2,504 samples and (2) the the first release of the phased UK Biobank which consists of 147,754 samples^10–12^. In each dataset, we filtered out monomorphic and multi-allelic variants and retained only data on chromosome 20. Management of the files containing the genotypes such as data filtering and subsampling was done using bcftools v1.15.1 (ref. ^20^). Number of samples, sample ancestries and number of variant sites can be found in Supplementary Table 1. We used the genetic map of chromosome 20 derived from the HapMap project^21^. For each genetic position of a reference panel not present in the genetic map, RESHAPE automatically infers the corresponding position in centimorgans using linear interpolation. As a null, we also generated an additional genetic map by keeping the HapMap size of the chromosome in centimorgans but assuming a constant recombination rate. We used RESHAPE to generate multiple synthetic reference panels using *K* = 1, 2, 4, 8, 16, 32 and 128. We also used the original reference panels, which we called *K* = 0.

To quantify the depletion of LD due to the introduction of recombination events in the data, we used LD scores. The LD score of a given variant is defined here as the sum of *r*^2^ between this variant and all other variants in its vicinity (±100 Kb). Global changes in LD scores were primarily assessed using Pearson’s correlation coefficient between the LD scores of the original and the synthetic reference panels. We complemented this by fitting linear-regression models using the LD scores of the synthetic and original panels as explanatory and dependent variables, respectively. With an *r*^2^ close to 1, any deviation from 1 in the regression slope (*β*) would reflect a global increase (*β* > 1) or decrease (*β* < 1) in LD scores.

To assess the reduction in imputation accuracy, we mimicked SNP array data by using genomes sequenced at high coverage. We masked genotypes at markers not present in three different SNP arrays (see below), then we imputed back these missing genotypes using the original and synthetic reference panels with BEAGLE v.5.3 (ref. ^2^) and finally compared the imputed genotypes to the high-coverage ones. We performed two imputation experiments based on the high-coverage sequencing data for 2,504 samples from the 1000GP (ref. ^22^) and for 147,754 samples from the UK Biobank^10–12^. In each dataset, we extracted a small number of samples to act as target samples and used the remaining ones as reference panel data, synthetic or not (Supplementary Table 1). We simulated two types of SNP array for experiments on the 1000GP: the Illumina HumanOmni 2.5 array and the Illumina Global Screening array. For the experiments on the UK Biobank, we used only the UKB Axiom array. The concordance between true and imputed genotype calls was computed using GLIMPSE_concordance v.1.1.1 (ref. ^4^).

### Reporting summary

Further information on research design is available in the Nature Portfolio Reporting Summary linked to this article.

### Supplementary information

Supplementary Information Supplementary Table 1, Figs. 1–5 and Methods.

Reporting Summary

### Source data

Source Data Fig. 2 Analysis of the effect of RESHAPE on linkage disequilibrium.

Source Data Fig. 3 Imputation accuracy with the 1000 Genomes Project.

Source Data Fig. 4 Imputation accuracy with the UK Biobank.

## Data Availability

The 1000 Genomes Project phase 3 dataset sequenced at high coverage by the New York Genome Center is available on the European Nucleotide Archive (ENA) under accession number PRJEB31736, the International Genome Sample Resource (IGSR) data portal and the University of Michigan school of public health ftp site (ftp://share.sph.umich.edu/1000g-high-coverage/freeze9/phased/). The publicly available subset of the HRC dataset is available from the European Genome-phenome Archive at the European Bioinformatics Institute (EBI) under accession number EGAS00001001710. The UK Biobank genetic data are available under restricted access. Access can be obtained by application via the UK Biobank Access Management System (https://www.ukbiobank.ac.uk/). Source data are provided with this paper.

## Code availability

The RESHAPE source code is available with MIT license from https://github.com/TheoCavinato/RESHAPE. RESHAPE can also be downloaded from Zenodo at 10.5281/zenodo.10970473 (ref. ^23^).
