# Supplementary material for: A resampling-based approach to share reference panels
Source: Nat Comput Sci. 2024 May 14;4(5):360–6. doi: 10.1038/s43588-024-00630-7 (PMC11136649; doi:10.1038/s43588-024-00630-7)
Supplement: Supplementary file 1 — Supplementary Table 1, Figs. 1–5 and Methods. [file 43588_2024_630_MOESM1_ESM.pdf]

nature computational science

Article

https://doi.org/10.1038/s43588-024-00630-7

A resampling-based approach to share reference panels

In the format provided by the authors and unedited

Supplementary Information

Table of Contents

Supplementary Table 1.
Description of the datasets. (page 1)

Supplementary Figure 1.
Time and memory required to generate a synthetic reference panel. (page 2)

Supplementary Figure 2.
Imputation accuracy on synthetic haplotypes generated with HAPGEN and RESHAPE. (page 3)

Supplementary Figure 3.
Imputation accuracy on synthetic haplotypes depending on the input genetic map. (page 3)

Supplementary Figure 4.
RESHAPE's pseudocode. (page 4)

Supplementary Figure 5.
Imputation accuracy on synthetic haplotypes depending on the population of the target sample. (page 5)

Supplementary Method.
Comparison of RESHAPE, HAPGEN and HAPNEST. (page 6)

Dataset	#Samples	#Variants after filtering	#Samples in reference panel	#Target samples	SNP array
European (EUR) samples from the 1000GP	503	110’708	These datasets were not use in imputation analysis		
African (AFR) samples from the 1000GP	661	138’699			
1000GP	2’504	2’116’846	2’452	52	Illumina Global Screening Array
					Illumina Human Omni 2.5 array
UK Biobank	147,754	13’677’164	146’754	1’000	UKB Axiom Array

Supplementary Table 1. Datasets, number of samples, variants after filtering, samples in the reference panel, target samples and names of the SNP-arrays used for each analysis

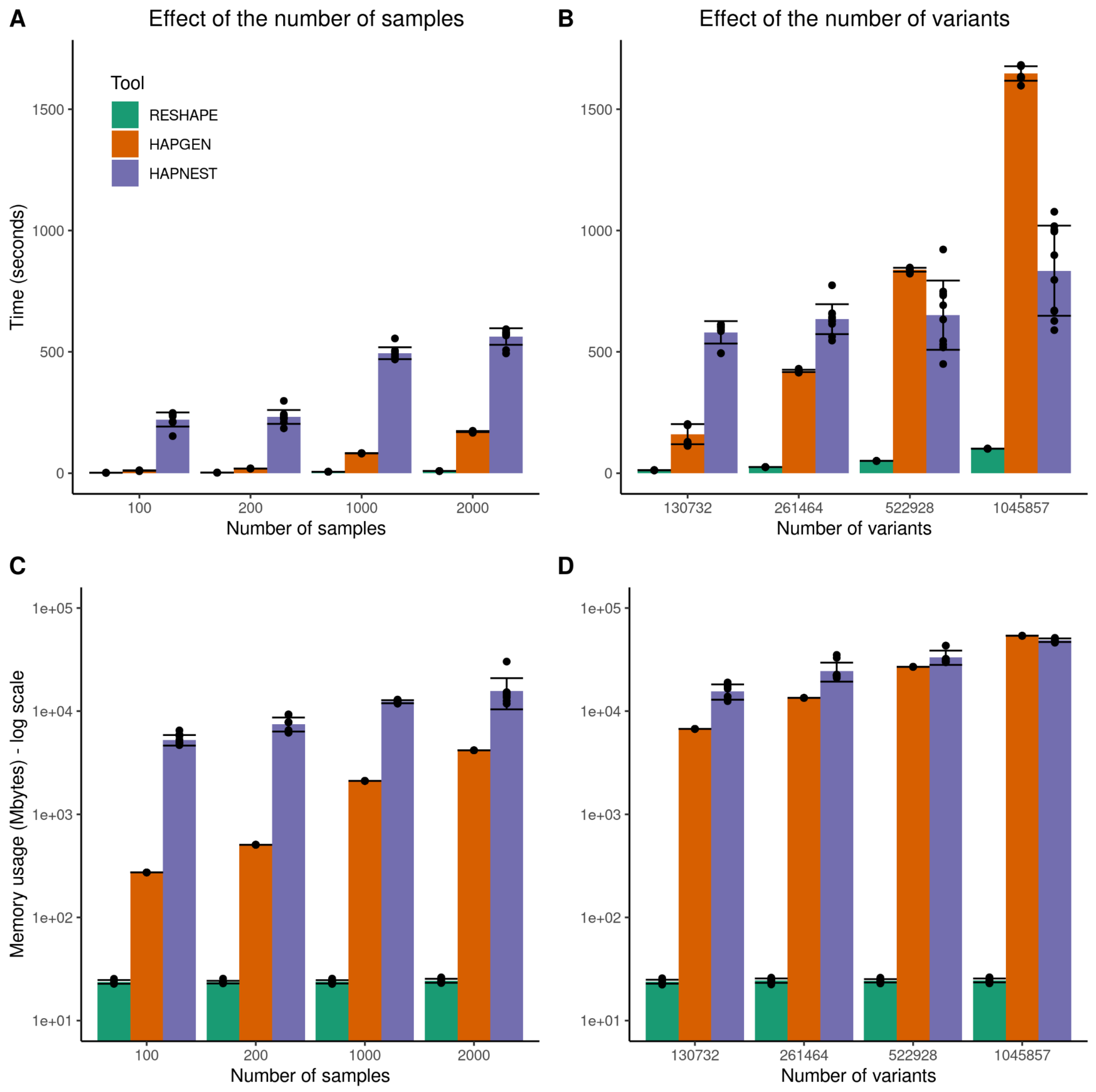

Supplementary Figure 1 | Time and memory required to generate a synthetic reference panel. Colors correspond to the method used to generate the synthetic reference panels. The top of each bar corresponds to the average time/memory required through 10 replicates. The error bars correspond to the mean of the 10 replicates +/- the standard deviation.

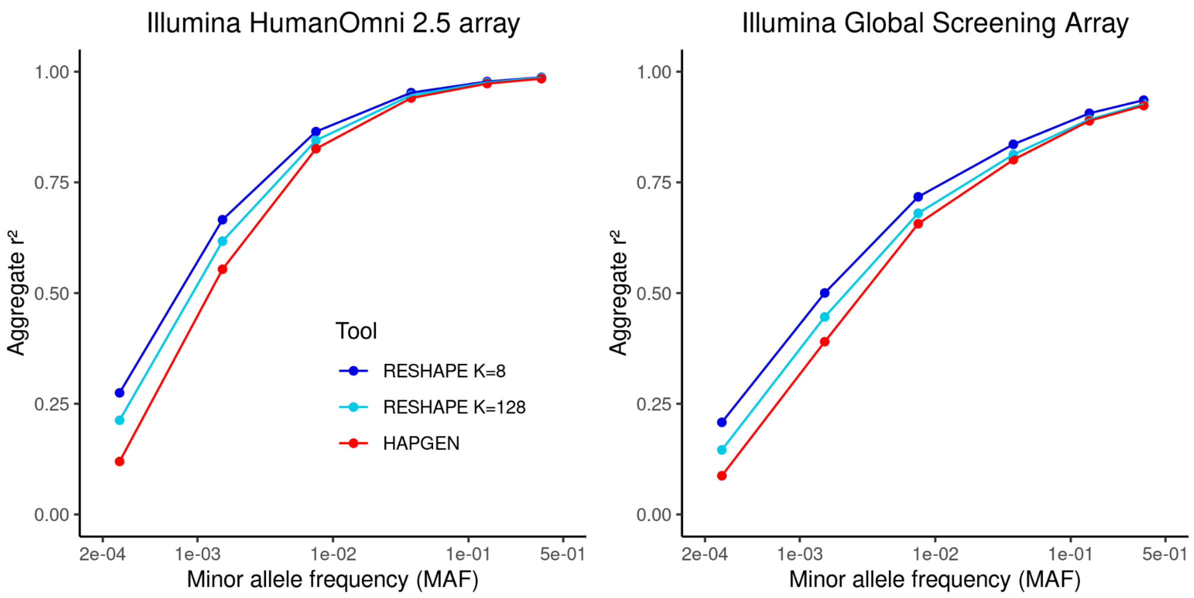

Supplementary Figure 2 | Imputation accuracy on synthetic haplotypes generated with HAPGEN and RESHAPE. Aggregate r² depending on the method used to generate a synthetic reference panel. Colors correspond to the method and parameters used to generate the synthetic reference panel.

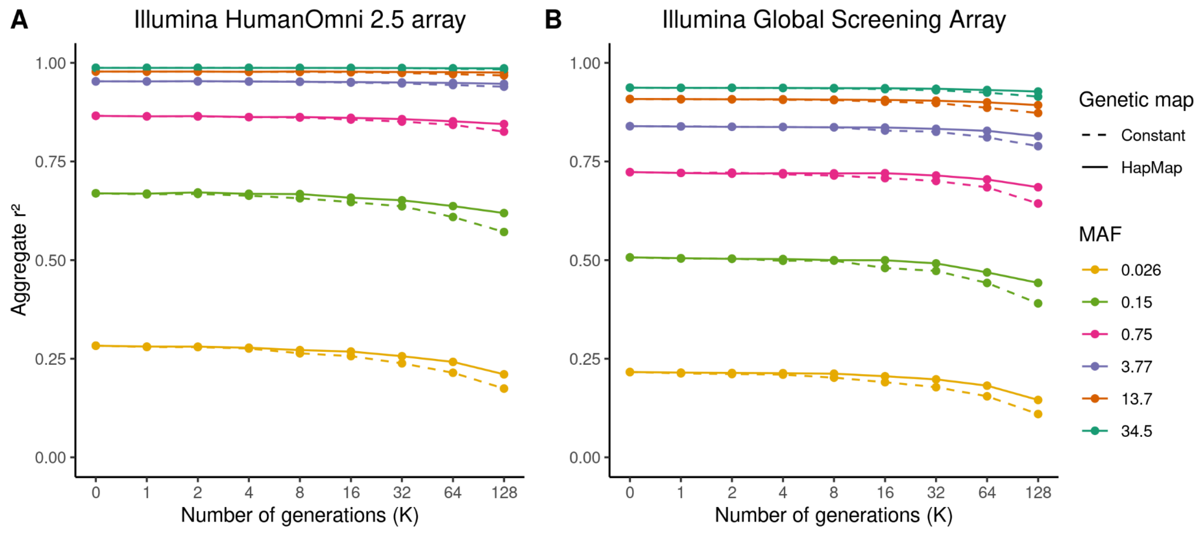

Supplementary Figure 3 | Imputation accuracy on synthetic haplotypes depending on the input genetic map. Aggregate r2 depending on the number of generations and genetic map used to recombine the haplotypes. Colors correspond to MAF bins. A) Results with the Illumina HumanOmni 2.5 array B) Results with the Illumina Global Screening Array.

```
Inputs:
    N: number of haplotypes in the reference panel
    K: desired number of meiosis
    V: variant call format file containing L variants
    G: genetic map

// 1st Step: Simulating recombination sites

chrSizeMorgan: size of the chromosome in Morgan (given by G)
R = [ ] // empty array to store simulated recombination events
λ = N/2 * K
r = 0

While r < chrSizeMorgan:
    U = uniformly sampled random value between 0 and 1
    D = -ln(1 - U) / λ // Inversed CDF-1 of a Poisson distribution
    r += D
    if r < chrSizeMorgan: convert r from Morgan to bp (using G) and append r to R

// 2nd Step: Permuting haplotypes based on simulated recombination events

H = [0..N-1] // indexation to follow to write haplotypes
randomly shuffle indexes in H

For each l in 0..L-1:
    p: position of V[ l ] in bp
    p-1: position of V[ l-1] in bp (if l=0 set p-1=0)
    For each r in R comprised between p and p-1:
        Permute two randomly sampled indexes in H
    Write V[ l ] while following haplotype order defined by H
```

Supplementary Figure 4 | Pseudocode for simulating recombination events and recombining the reference panel accordingly.

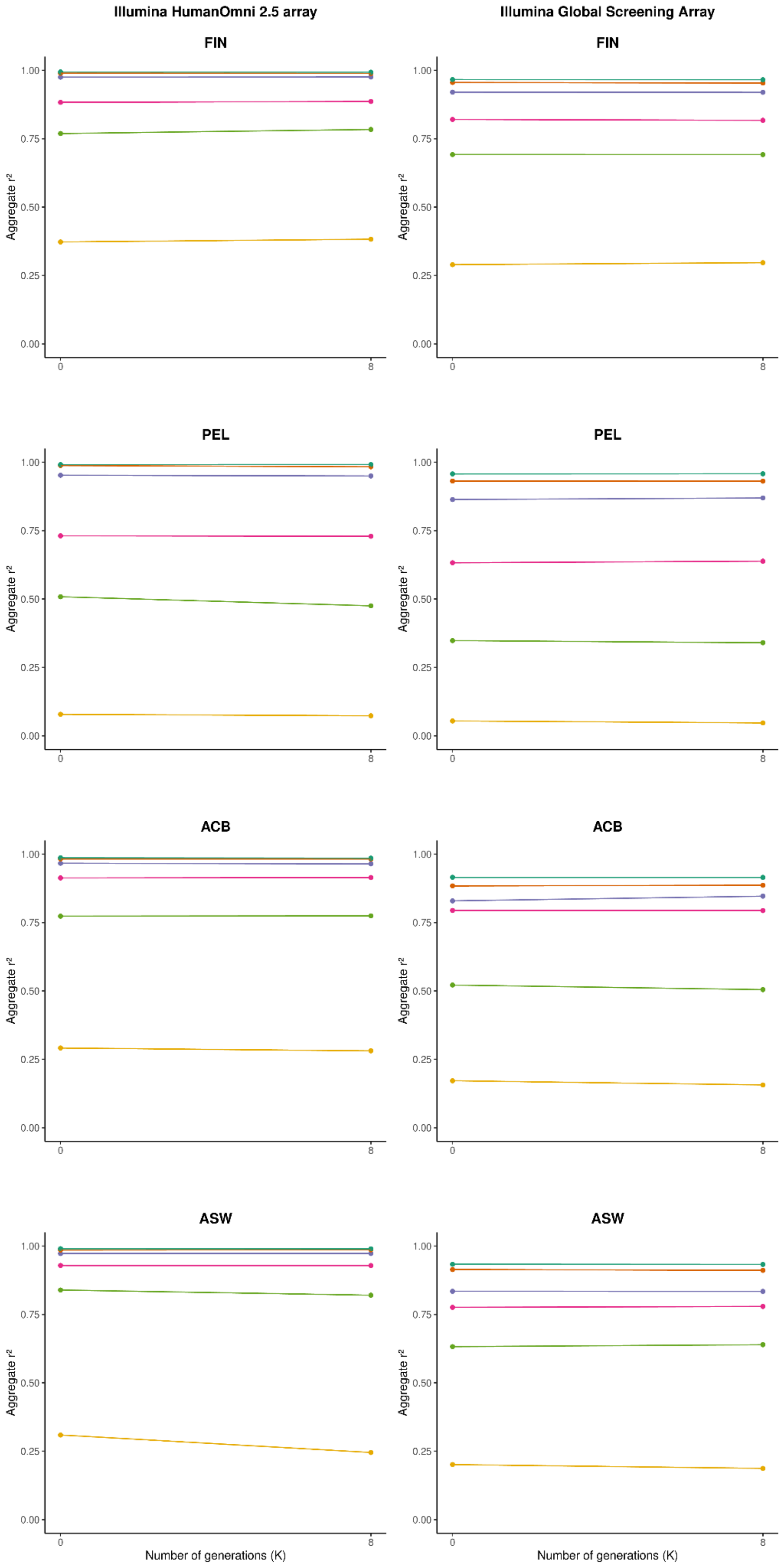
Illumina HumanOmni 2.5 array
Illumina Global Screening Array
FIN
PEL
ACB
ASW
Aggregate r²
Number of generations (K)
0.00
0.25
0.50
0.75
1.00
0
8

Supplementary Figure 5 | Imputation accuracy on synthetic haplotypes depending on the population of the target sample. Aggregate r² depending on the number of generations (K=0 or K=8) for target individuals from different populations. Colors correspond to MAF bins. The results obtained with the Illumina HumanOmni 2.5 array are displayed on the left and the results with the Illumina Global Screening Array are displayed on the right.

Supplementary Method.

1. Time and memory

To compare the speed and memory consumption of our approach against HAPGEN and HAPNEST we generated synthetic reference panels with different numbers of variants (130’732, 261’464, 522’928 and 1’045’857) and samples (100, 200, 1000 and 2000). The datasets used to assess the effect of the number of samples contained 130’732 variants and the datasets used to assess the effect of the number of variants contained 2452 samples. These datasets were generated using the chromosome 20 of the 1000GP dataset described in more detail in the main method.

To generate a synthetic reference panel of the same size as the input reference panel, we set the number of samples to generate equal to the number of samples present in the input dataset using the -n parameter in HAPGEN and the *nsamples* parameter in HPANEST. We only simulated control samples and selected a random genetic position for the -dl parameter when using HAPGEN. When allowed by the method, we set the number of threads to 8. All other parameters were set to default for both methods. For the recombination maps we used the genetic map of chromosome 20 derived from the HapMap project.

To compute the time and memory required by each algorithm we used the bash “time” command with the -v option. The total running time on **Supplementary Figure 1** is the sum of the fields “System time (seconds): 0.00” and “Percent of CPU this job got: 100%”. The memory usage corresponds to the field “Maximum resident set size (kbytes)”.

We ran each analysis 10 times and the height of each bar in **Supplementary Figure 1** corresponds to the mean of these 10 replicates and the error bar to the mean +/- the standard deviation.

2. Imputation accuracy

In the imputation accuracy analysis, we replicated the method described in the main Method section when using RESHAPE but only for K=8 and 128. For HAPGEN, we replicated the same pipeline but using HAPGEN with default parameters to simulate the synthetic reference panels.
