# Supplementary material for: A resampling-based approach to share reference panels
Source: Nat Comput Sci. 2024 May 14;4(5):360–6. doi: 10.1038/s43588-024-00630-7 (PMC11136649; doi:10.1038/s43588-024-00630-7)
Supplement: Supplementary file 2 — Reporting Summary [file 43588_2024_630_MOESM2_ESM.pdf]

nature portfolio

Corresponding author(s): Olivier Delaneau

Last updated by author(s): Apr 17, 2024

Reporting Summary

Nature Portfolio wishes to improve the reproducibility of the work that we publish. This form provides structure for consistency and transparency in reporting. For further information on Nature Portfolio policies, see our Editorial Policies and the Editorial Policy Checklist.

Statistics

For all statistical analyses, confirm that the following items are present in the figure legend, table legend, main text, or Methods section.

n/a	Confirmed	
☐	☒	The exact sample size (*n*) for each experimental group/condition, given as a discrete number and unit of measurement
☒	☐	A statement on whether measurements were taken from distinct samples or whether the same sample was measured repeatedly
☒	☐	The statistical test(s) used AND whether they are one- or two-sided<br>*Only common tests should be described solely by name; describe more complex techniques in the Methods section.*
☒	☐	A description of all covariates tested
☐	☒	A description of any assumptions or corrections, such as tests of normality and adjustment for multiple comparisons
☐	☒	A full description of the statistical parameters including central tendency (e.g. means) or other basic estimates (e.g. regression coefficient) AND variation (e.g. standard deviation) or associated estimates of uncertainty (e.g. confidence intervals)
☒	☐	For null hypothesis testing, the test statistic (e.g. *F*, *t*, *r*) with confidence intervals, effect sizes, degrees of freedom and *P* value noted<br>*Give P values as exact values whenever suitable.*
☒	☐	For Bayesian analysis, information on the choice of priors and Markov chain Monte Carlo settings
☒	☐	For hierarchical and complex designs, identification of the appropriate level for tests and full reporting of outcomes
☐	☒	Estimates of effect sizes (e.g. Cohen's *d*, Pearson's *r*), indicating how they were calculated

Our web collection on statistics for biologists contains articles on many of the points above.

Software and code

Policy information about availability of computer code

Data collection	We did not use software for the data collection.
Data analysis	The analyses were made using the following publicly available softwares:<br><br>bcftools v1.15.1 (genotype management)<br>BEAGLE v.5.3 (imputation method)<br>GLIMPSE concordance v1.1.1 (imputation accuracy)<br>HAPGEN v2.2.0 (comparison)<br>HAPNEST v1.0.0 (comparison)<br>RESHAPE v1.0.0 (tool presented in the article): https://github.com/TheoCavinato/RESHAPE

For manuscripts utilizing custom algorithms or software that are central to the research but not yet described in published literature, software must be made available to editors and reviewers. We strongly encourage code deposition in a community repository (e.g. GitHub). See the Nature Portfolio guidelines for submitting code & software for further information.

Data

Policy information about availability of data

All manuscripts must include a data availability statement. This statement should provide the following information, where applicable:

- Accession codes, unique identifiers, or web links for publicly available datasets
- A description of any restrictions on data availability
- For clinical datasets or third party data, please ensure that the statement adheres to our policy

The 1000 Genomes Project phase 3 dataset 398 sequenced at high coverage by the New York Genome Center is available on the European Nucleotide Archive (ENA) under accession no. PRJEB31736, the International Genome Sample Resource (IGSR) data portal and the University of Michigan school of public health ftp site (URL: ftp://share.sph.umich.edu/1000g-high-coverage/freeze9/phased/).
The publicly available subset of the HRC dataset is available from the European Genome-phenome Archive at the European Bioinformatics Institute (EBI) under accession no. EGAS00001001710.
The UK Biobank genetic data are available under restricted access. Access can be obtained by application via the UK Biobank Access Management System (URL: https://www.ukbiobank.ac.uk/).

Human research participants

Policy information about studies involving human research participants and Sex and Gender in Research.

Reporting on sex and gender	We did not perform sex- or gender-based analyses
Population characteristics	We compared the imputation accuracy on synthetic haplotypes generated with RESHAPE depending on the population of the target samples from the 1000 genomes project.
Recruitment	N/A
Ethics oversight	N/A

Note that full information on the approval of the study protocol must also be provided in the manuscript.

Field-specific reporting

Please select the one below that is the best fit for your research. If you are not sure, read the appropriate sections before making your selection.

☒ Life sciences ☐ Behavioural & social sciences ☐ Ecological, evolutionary & environmental sciences

For a reference copy of the document with all sections, see nature.com/documents/nr-reporting-summary-flat.pdf

Life sciences study design

All studies must disclose on these points even when the disclosure is negative.

Sample size	We report the samples sizes used for each data sets in Supplementary Table 1. The sample size correspond to the maximum number of samples we had access to. When using samples from a same population we used all the samples of this population in the thousand genome project. The 52 samples used for imputation from the thousand genome project correspond to 2 randomly selected samples from each of the 26 population in the thousand genome project.
Data exclusions	No data was excluded.
Replication	The softwares and the data used in this article are publicly available. The main conclusions we made based on our on analysis performed with the thousand genomes project holds for the analysis we made with the UK Biobank. We made available the version of the softwares we used to generate our results so that other researchers can reproduce our experiments.
Randomization	We did not randomize since there are no experimental groups in this study.
Blinding	No group allocation occurs in this study.

Reporting for specific materials, systems and methods

We require information from authors about some types of materials, experimental systems and methods used in many studies. Here, indicate whether each material, system or method listed is relevant to your study. If you are not sure if a list item applies to your research, read the appropriate section before selecting a response.

Materials & experimental systems

n/a	Involved in the study
☒	☐ Antibodies
☒	☐ Eukaryotic cell lines
☒	☐ Palaeontology and archaeology
☒	☐ Animals and other organisms
☒	☐ Clinical data
☒	☐ Dual use research of concern

Methods

n/a	Involved in the study
☒	☐ ChIP-seq
☒	☐ Flow cytometry
☒	☐ MRI-based neuroimaging
